# Improving Case-Based Reasoning Systems by Combining *K*-Nearest Neighbour Algorithm with Logistic Regression in the Prediction of Patients’ Registration on the Renal Transplant Waiting List

**DOI:** 10.1371/journal.pone.0071991

**Published:** 2013-09-09

**Authors:** Boris Campillo-Gimenez, Wassim Jouini, Sahar Bayat, Marc Cuggia

**Affiliations:** INSERM U936, University of Rennes 1, Brittany, France; UC Davis School of Medicine, United States of America

## Abstract

**Introduction:**

Case-based reasoning (CBR) is an emerging decision making paradigm in medical research where new cases are solved relying on previously solved similar cases. Usually, a database of solved cases is provided, and every case is described through a set of attributes (inputs) and a label (output). Extracting useful information from this database can help the CBR system providing more reliable results on the yet to be solved cases.

**Objective:**

We suggest a general framework where a CBR system, viz. K-Nearest Neighbour (K-NN) algorithm, is combined with various information obtained from a Logistic Regression (LR) model, in order to improve prediction of access to the transplant waiting list.

**Methods:**

LR is applied, on the case database, to assign weights to the attributes as well as the solved cases. Thus, five possible decision making systems based on K-NN and/or LR were identified: a standalone K-NN, a standalone LR and three soft K-NN algorithms that rely on the weights based on the results of the LR. The evaluation was performed under two conditions, either using predictive factors known to be related to registration, or using a combination of factors related and not related to registration.

**Results and Conclusion:**

The results show that our suggested approach, where the K-NN algorithm relies on both weighted attributes and cases, can efficiently deal with non relevant attributes, whereas the four other approaches suffer from this kind of noisy setups. The robustness of this approach suggests interesting perspectives for medical problem solving tools using CBR methodology.

## Introduction

Case-based reasoning (CBR) is a problem-solving paradigm emerging in medical decision-making systems [1]. Instead of relying solely on general knowledge of a problem domain, CBR utilizes the specific knowledge of previously experienced, concrete problem situations - also referred to as *cases* - to tackle new ones [2]. More specifically, CBR methodology defines a general *CBR cycle* composed of four steps centered around a case database [3]. First, the decision making process needs to identify, among the solved cases, those that seem to be the most similar to the considered unsolved case. Then, solve the new case relying on the knowledge extracted from the most similar solved cases. The third step consists in evaluating the suggested solution for the new case. Finally, if the solution is found satisfactory, the decision making process usually stores the part of the experiment likely to be useful for future problem solving. CBR in biology and medicine has found one of its most fruitful application areas and appears particularly suited to designing decision making tools in the field of Health sciences [4]. Indeed, Medicine appears as a highly intensive-data field where it is advantageous to develop systems capable of reasoning from pre-existing cases such as from electronic health record repositories for instance.

The present paper focuses on the two first steps of the CBR cycle, viz. retrieve and reuse solutions from previously experienced situations, called cases. Each case is a problem description linked to its solution. For solving new problems, the decision making process requires to select relevant cases, by measuring similarity of common characteristics between the new and the previously experienced cases [5]. In accordance with the traditional CBR view, the knowledge database contains cases, which consist in a problem-specific definition and construction. Thus, there are as many case bases as problems to be solved. Bergmann *et al.* overcome that problem by introducing the concept of utility [6]. Similarity measures are not directly computed from the problem descriptions of new and previously experienced cases, they are computed with the description of their utility; utility description being specifically defined in accordance with the solution needed.

Statistical analyses and regression modeling could be useful to introcuce utility description in CBR systems, by converting medical data sources *- or data bases -* into medical case bases. Regression models contain a part of knowledge which may be used to define utility description of cases and to perform problem-specific measures of similarity. The paper precisely consists of such an illustration by the formal definition of a traditional CBR retrieval algorithm ‘*the K-Nearest Neighbour (K-NN) algorithm’* coupled with a logistic regression model, and its comparison with the regression model and the *K*-NN algorithm alone for the prediction of registration on the renal transplant waiting list.

## Materials and Methods

### 2.1 Domain Application and Data Source

To carry out this work, we used data from the French Renal Epidemiology and Information Network (REIN) registry [7] related to renal replacement therapies (RRT) for end-stage renal disease (ESRD), and data from the *Agence de la Biomédecine*, the French national agency of organ transplantation for registration on the waiting list of kidney transplantation.

Registration on the waiting list is a medical decision based on medical factors in accordance with French medical guidelines that do not really need automated decision-making support. Nevertheless, those data and their domain application were chosen for several reasons:

- Data come from a national registry that confirms the data quality by the French *Comité National des registres* agreement.
- Many studies showed that the selection criteria on the waiting list diverge from one center to another, and that access to the renal transplant waiting list is influenced by both medical and non medical factors [8].
- Recent studies showed that it is possible to predict access to the waiting list relying on some of these factors [9], [10].
- Our main objective is a methodological essay on combination of CBR retrieval algorithm with logistic regression, and not the implementation of a medical decision support.

### 2.2 Study Population and Data Collection

The study population consists of every incident ESRD patients in Brittany, limited to those who started an RRT (peritoneal dialysis or hemodialysis) between January the 1st, 2004 and December the 31th, 2008. Patients who received a preemptive transplant and patients who came back on the waiting list after a first transplant have been excluded.

The dependent variable for the study was the patients registration on the renal transplant waiting list *(e.g. registered on the waiting list: yes/no)*. The registration status was computed relying on the date of the first RTT as well as the date of registration on the waiting list. Only patients recorded on the waiting list within 12 months after inclusion on the REIN registry have been considered as registered patients.

A set of description factors have been defined according to data availability of the REIN database and the renal transplant scientific literature [8], [11]–[14]. All factors have been reduced to a binary value in order to simplify similarity calculation, in accordance with the traditional dichotomization procedure retrieved in the literature [8]. Three categories of factors likely to be related to registration on the transplant waiting list have been studied:

- Social and demographic factors: sex, age and current occupation at the first RRT.
- Clinical and biological factors at the first RRT: existence of hypertension, diabetes, chronic respiratory failure, chronic heart failure, ischemic heart disease, heart conduction disorder or arrhythmia, positive serology (HCV, HBV, HIV), liver cirrhosis, disability, past history of malignancy and hemoglobin as <11 g/dl and ≥11 g/dl.
- Factors related to medical care: ownership of nephrology facility where the first RRT were performed (private or public), follow-up in institution performing transplantation, type of first RRT (hemodialysis or peritoneal dialysis), urgent versus planned first dialysis session and first catheterization.

Due to missing data (≥10%), some factors potentially related to registration on the waiting list have not been considered either for statistical analyses or CBR algorithms: distance from patients residence to the transplantation department, smoking status, body mass index, vascular comorbidities and serum albumin level.

### 2.3 Decision Making Model

#### 2.3.1 Decision making process and mathematical notations

We depict, in this section, the overall mechanism designed to predict patient accessibility to renal transplant waiting list. Upper case notations refer to vector (or a set of vectors, viz., a matrix) whereas lower case notations refer to scalar real variables -an exception is made for the scalar parameter *K* of the *K*-NN algorithm for the sake of consistency with the literature. Curved notations denote sets of elements. For the sake of generality, Let 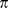 refer to the decision making process considered hereafter. Moreover, let 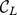 refer to a set of labeled cases, viz. patients, and let 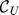 refer to a set of new analyzed cases. We aim at designing a decision making process that maps new cases to previously solved (i.e., labeled) cases.

We consider two possible classes: as a matter of fact, a patient is either registered in the renal transplant waiting list or not. Consequently, the labels are assumed to be binary. let 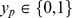 denote the label assigned to patient 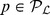, where 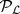 refers to the set of patients considered in 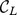. The set of *cases* consists, in either case-sets -labeled, 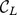, or not 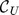
- of a set of patients, 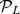 (or 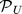 respectively), and two sub-sets: 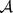 and 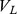 (or 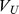 in the case of 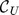) named respectively, *Attribute*-set and *Value*-set. On the one hand, 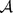 represents the set of elements that characterize a case such as, social and demographic data (e.g., age, gender and current occupation for instance) and, clinical as well as biological data (e.g., existence of hypertension, diabetes, chronic respiratory failure, chronic heart failure, to name a few). The complete set of criteria is further detailed in the section 0.2. The set 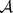 is considered common to both 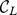 and 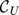. On the other hand, 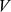 (i.e., either one of the sets 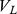 and 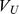) represents a set of vectors related to the considered attributes for every patient: Let, 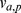 refer to the value assigned to the attribute 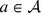 for the patient 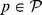 (i.e., either one of the sets 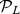 and 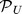). For the sake of ease of representation, 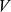 can be seen as a matrix of size 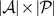 (*the product of the cardinal of both sets 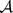 and* 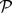), where every cell contains a value 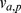. For every attribute 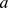, a patient 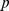, can either verify the attribute 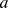 or not. Consequently, 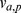 can only take a binary value in 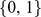, where 1 refers to *attribute verified* and 0 otherwise. Thus, 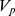 refers to a vector of 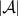 binary elements that represents the condition of a patient 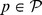 regarding a set of attributes 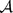. As previously mentioned, the set of patients 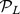 considered in 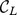 is already labeled. The set of labels 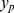 are stored in a vector 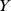.

Finally, we can see the decision making process 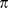 as a function that classifies unlabeled patients in the set 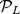 relying on the similarity of the unlabeled patients with the set of labeled patients. Let 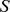 refer to the vector of labels provided by the decision making engine, where every patient 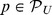 is assigned a numerical value 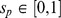, such that for every patient 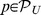:
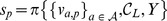
(1)where 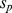 quantifies the possible proximity of patient 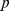 to the possible classes in 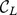. If 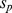 is a binary value, i.e. 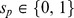, the decision making policy 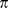 is referred to as a hard classification. Otherwise, it is usual to speak of soft classification. We consider in this paper this latter approach.

In the context of CBR, the decision maker assigns a label to new cases depending on their similarity with previously solved cases. The assignment relies on a measure that quantifies the resemblance of the analyzed case with the set of labeled cases. Such decision making approach mimics the decision making process of a physician when dealing with new patients for instance. To do so, the decision maker needs to assess the importance of the different factors as well as the reliability of the cases, i.e. patients, dealt with in the past. In this paper, the designed CBR relies on a soft 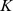-NN algorithm, perhaps one of the most widely used technology in CBR [15]. Namely, rather than assigning a label to either classes, we compute a probability of being assigned such labels. Such probability is computed relying on the 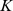 most similar patients already labeled. A simple threshold decision making would lead to a hard classification process.

Designing our decision making mechanism requires estimating the distance between patients as well as qualifying the reliability of the labeled patients. These notions are discusses in the next sections.

#### 2.3.2 Similarity metric and attributes' weights

Ideally speaking, similar patients should belong to a same class (registered or not registered). Similar patients usually express similar values to their respective attributes. Equivalently, to the notion of similarity, we can define a distance measure that quantifies the proximity of the new patient to treat with the previously seen patients (i.e. the labeled set of patient). The larger the similarity measure is the smaller becomes the distance.

For the sake of simplicity we define, in this paper, the distance measure as follows. Let 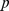 and 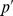 denote two patients (label or unlabeled), the distance between these patients is quantified through the measure:
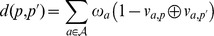
where 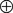 refers to the exclusive OR (XOR) operator and such that:



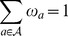
where, 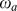 denotes the weight assigned to attribute 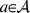, and the similarity measure appears equal to:



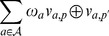



The weights 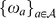 are, usually, not known *a priori*. Therefore, the decision maker needs to acquire that information through a learning process. Thus, relying on the labeled set of cases, the decision maker estimates the impact of the various attributes considered. This step is discussed in Section 0.4, where all required learning steps are detailed.

#### 2.3.3 Soft 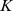-Nearest neighbour algorithm


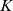
-NN Algorithms refer to simple classification techniques that assign labels to new cases depending on their similarity with a reference set of already labeled cases. Thus, for every new patient 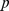 to label, 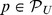, a 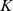-NN algorithm operates through mainly two major steps, the selection step and the fusion step.

1. *Selection Step:*

- Compute first the similarity of patient 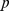 with patients 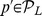.
- Sort the similar patients 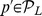 according to their similarity measure.
- Select the 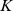 most similar patients 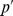.

1. *Fusion Step:* Compute a numerical value that quantifies the proximity of the new case (i.e. Patient 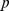) to the set of possible classes in the training set (i.e. 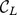).

Depending on this last step, a decision maker can, if needed, assign a label to the new case. Usually a threshold based classifier is used for the assignment process. This latter is however out of the scope of this paper.

Let 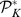 refer to the optimal 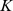-NN set obtained after the selection step. More specifically 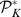 contains the K labeled patient -stored in 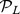
- that have the largest similarity measures with respect to the currently analyzed patient 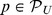. The fusion step consists in quantifying the possible outcome of the decision making process. Finally, the outcome of the decision making process, 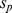 for a patient 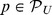 is defined as:
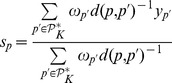
(2)where the set of patients’ weights is denoted by the variables 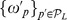, and 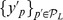 are the labels assigned to the labeled cases as defined in the section 0.3.1. The weights 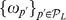 are designed to verify:



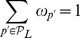



We conclude this section discussing, briefly, the settings of the 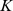-NN model: i.e., the selection of an appropriate value 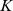. Usually, it is not possible to define, *a priori*, the value of the parameter 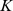. Thus, a setting phase is necessary to evaluate a satisfactory value with respect to a learning set. The setting phase consists in three steps. First, a specific subset 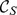 of the learning set 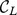, 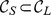, is defined. We refer to this subset as *setting set* in Section 0.5. Then an evaluation metric that quantifies how well behaves the 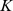-NN algorithm on the setting set is computed for the integers (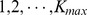) smaller than a specified limit 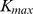. Finally the smallest integer 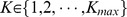 that maximizes the evaluation metric is kept and used on the set 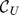 during the learning process.

### 2.4 Learning Process based on Logistic Regression

This section deals with the learning phase. As a matter of fact, in order to implement the 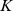-NN based CBR, we need to compute, on the one hand, the parameters 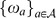 to evaluate the similarity between patients, and on the other hand, the parameters 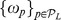 in order to evaluate the importance -or contribution- of each patient in 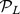. We consider the scenario where the set of parameters is computed once relying on the labeled cases. Then they are exploited to solve new cases.

#### 2.4.1 Logistic regression

In a nutshell, logistic models are useful to predict the presence or absence of an outcome or a characteristic based upon the values of a set 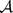 of predictor variables. The methods fits regression model for binary response data relying on the maximum likelihood method [16]. More specifically, in this paper we consider the following definition:

#### Definition 1 (Logistic regression)

*Let 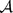 denote a set of explanatory variables, 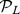 a set of cases, 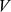 a binary matrix in 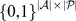 such that 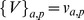 with 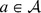 and 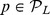, and finally, let 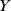 refer to a vector of binary expert outcomes (e.g., registered or not registered). LR assumes that there exist an underlying model that can explain the decision outcomes 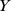 as a logistic function of the matrix 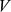 and a vector of regression parameters 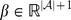. Then LR fits the data in 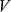 to a logistic function such that for any case 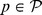 characterized by a vector of values of the set 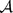:*

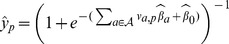

*where 
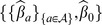
 represent maximum likelihood estimated regression parameters and 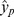, in 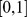 the estimated prediction outcome for any analyzed case 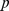
*.

In Definition 1, the regression coefficients reflect the relative influence of predictor factors to define cases’ registration on the waiting list. Thus it is natural to take them into account when computing the weights of the attributes 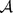 and the patients 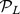 as described in Section 0.3. This matter is further detailed in next section.

#### 2.4.2 Weighting of attributes and patients

Significance of each factor, when the regression provides maximum likelihood estimates, could be based on the Wald’s test defined as follows:

**Definition 2 (Wald Statistic and Weighting of Attributes)** *Let 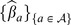 denote a vector of maximum likelihood estimates and 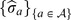 their respective maximum likelihood standard deviations. Then Wald’s statistic with respect to the attribute 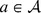 is defined as:*

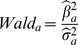



*Finally, the vector of weights of attributes, 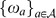, is defined such that:*

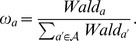



When dealing with the set of labeled cases 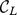, LR introduces a gap between the stored binary outcomes 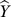 and the predicted soft outcomes . For every 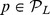, the value of the gap equals 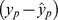. Relying on the definition of Pearson residuals, we introduce the cases’ attributes 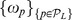 as follows:

**Definition 3 (Weighting Cases)** *Let 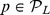 denote a labeled case, 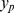 its label and 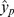 the logistic regression outcome. Pearson residuals are defined as:*

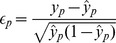
where 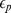 is assumed to be roughly drawn from a standard normal distribution. Thus 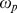 is defined as:
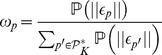

*where 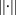 refers to the absolute value function and 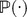 refers to the probability of observing 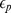, and thus to the density function of a standard normal distribution.*

We end this section introducing a last notation for the sake of clarity. Usually, many training phases are needed in order to estimated all the parameters of a complete decision making process. In such case, the labeled set 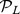 needs to be divided and distributed among the different phases. In this paper, the parameters of both the LR and the 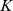-NN algorithm need to be learned. Thus the set 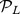 needs to be subdivided into two sets 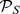, introduced in previous section, for the sake of the algorithm 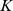-NN, and a set 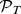, referred to as *training set*, dedicated to the estimates of LR parameters. Finally, 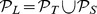 and since 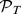 and 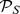 must not overlap, i.e., they contain no common cases We can write, to conclude this section, that their intersection is empty: 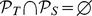.

## Experimental Protocol and Results

### 3.1 Data description: Training, Setting and Evaluating sets

The initial population included 1647 patients who began an ESRD treated by dialysis (652 (41%) women and 995 (60%) men). Among them, 350 i.e., 21%, have been registered on the waiting list of renal transplantation in the first year following the start of RRT.

Unfortunately, patients’ data with respect to the selected explicative variables (Cf section 0.2 for further details), were not always complete or fully available. Since, logistic models cannot deal with missing data, we decided to restrict this analysis to a subset of patients with no missing data. Thus, the study population was reduced to 1137 patients with complete data, which only represent 70% of the initial population. It is worth mentioning that the general caracteristics of this population remain similar to the original population. As a matter of fact, the population still included a majority of men (692 men, 61%) and the rate of patients registered on the waiting list remains similar to the original population (255 patients, 23%). For the rest of this section, we only focus on the 1137 patients with complete data. We denote this set of patients 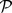 as introduced in previous sections. Thus, the set of patients 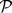 is such that 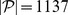. For the sake of the experiment, 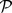 is distributed into two sets: 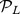 and 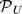. On the one hand, the set 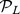 represents the labeled set that we use for training the LR as well as for setting the parameter 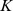 of the 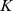-NN algorithm, while on the other hand, we kept a set 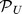, considered as the unlabeled data on which we apply our methodology, for the evaluation phase. The labeled set is also partitioned into two sets: 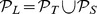. The training set 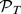 is dedicated to the LR, while the setting set 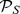 is used to estimate an appropriate 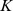-value of the 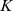-NN algorithm.

Finally, the training database, the setting database and the evaluation database are built relying on a random sampling for the set population set, such that:
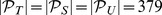



It is worth mentioning that no specific filtering was used to obtain the same number of patients in all three databases. It is a simple coincidence that occurred after discarding patients with incomplete data. A Pearsons chi-squared test was performed to verify that all three sets share common characteristics. The chi-squared test showed no significant difference between the three databases (data not shown).

### 3.2 Experimental Protocol

The key aims of this section are twofold. On the one hand, we describe the algorithms considered in this experimental section and compare them to the overall approach detailed hereabove. On the other hand, we present the evaluation criteria considered in this paper to assess the quality of the different simulated approaches.

As discussed in previous Sections, we consider in this paper the combination of a case based reasoning approach, viz. *K*-NN algorithm, with a logistic regression model. Moreover, in order to enhance its behavior, we suggested several weighing parameters that capture the relevance of the explicative variables and the labeled cases. In order to evaluate the suggested approach, we propose to simulate five different algorithms analyzed within two scenarios. The five algorithms combine different elements described in Sections 0.3 and 0.4. First we simulate, separately, the two main algorithms describes in previous sections:

- (i) The standalone logistic regression algorithm.
- (ii) The standalone *K*-NN algorithm (also referred to standalone CBR algorithm in the rest of the paper).

Both algorithms were extensively studied and know to be efficient prediction tools. In order to analyze the benefit of weighting the attributes and/or the patients, we start by simulating the standalone versions. Then we progressively add the weighting variables introduced in sections 0.3.2 and 0.4.2. This results into three other approaches to consider. Thus, we can enumerate the following algorithms:

- (iii) A *K*-NN with weighted attributes (also referred to as *CBR+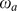
* in the simulation results).
- (iv) A *K*-NN with weighted patients (also referred to as *CBR+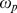
* in the simulation results).
- (v) A *K*-NN with both weighted attributes and weighted patients (also referred to as *CBR+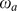
+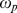
* in the simulation results). This latter is the suggested approach of this paper. The four other algorithms are used as comparison material.

All five algorithms are computed within two scenarios: on the one hand, 19 explicative variables, i.e., attributes, that comply with the general medical model are used. This first scenario analyses the performances of these algorithms when the variables are already reliable from the empirical point of view. On the other hand, 50 additional attributes randomly defined are considered in the second scenario in order to evaluate the robustness of the simulated algorithms with respect to uncertain models. Namely, the objective is to study the behavior of the prediction tools when the knowledge database contains factors not related to the prediction object.

**Figure 1:**
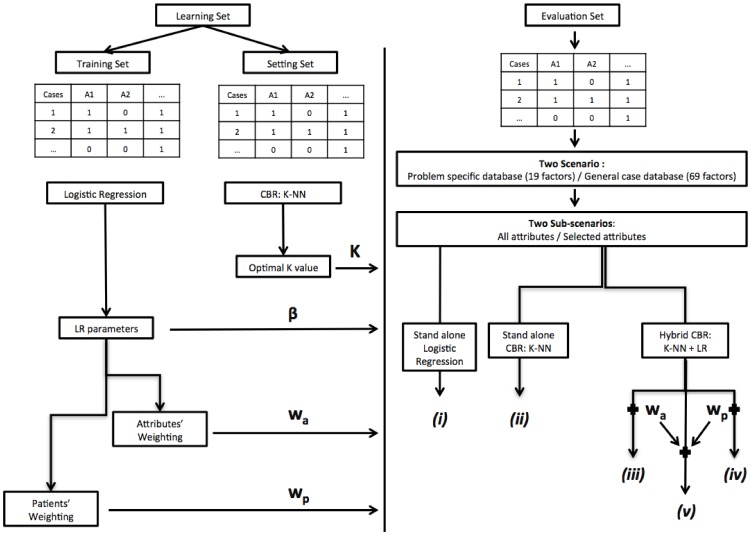
Experimental Protocol. During the learning phase, a training set is used to compute the parameters of a logistic regression model. These parameters enable the computations of the weights of attributes as well as patients’ weights. Then a setting set is used to evaluate an optimal *K* value for the *K*-NN algorithm. Finally all these estimates are exploited to evaluate five decision making algorithms referred to by the indexes *(i)* to *(v)*.

Moreover, in every scenario we evaluate the benefit of automated variable selection for LR before simulating the algorithms. Thus for every scenarios, we describe two sub-scenarios. We refer to them in simulations as the sub-scenarios *Prediction using all attributes* and *Prediction using selected attributes*. All scenarios and algorithms are summarized in Figure 1.

All performance results are presented in terms of the receiver operating characteristic curve (AUC). In order to compute confidence intervals of AUC results, a bootstrap resampling procedure is performed [17]. Thus, the probability distribution of AUC statistic is simulated by 500 random samples from the original evaluation database. Then a specific non parametric Monte Carlo AUC estimator, 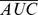, is computed. The chosen estimator is a non biased AUC estimator such that:
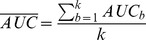
where the index 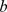 refers to the bootstrap iteration and 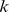 is the total number of iterations (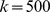 in this case). We computed the performance evaluation estimates such that the confidence intervals limits are the 2.5 and 97.5 percentiles of the 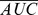 distribution.

### 3.3 Computational Tools

All computations involved in this study, including LR and CBR algorithms, were performed on the free software environment ‘R’ version 2.12.2 GUI 1.36 Leopard build 32-bit for Mac OS X [18].

More specifically, we relied on the package ‘stats’ (version 2.12.2) to implement logistic regression. As a matter of fact, it allows modeling generalized linear models thanks to the ‘glm’ function. Then, the functions ‘Anova’ and ‘summary’ enabled the estimates of our LR parameters. Finally, the ‘step’ function was used to perform a backward stepwise selection of the LR variables relying on Akaike’s criterion. Related to CBR algorithms, we designed our specific functions using the programming language of the R user interface to ensure calculation of similarity measures, selection of nearest neighbours, prediction of probability to be registered, and classification of cases.

### 3.4 Results

Table 1 shows the weights of attributes calculated from the Wald statistics using the regression coefficient estimates of the LR, as defined in section 0.4.2, and their respective standard deviations. Both sub-scenarios, summarized in Figure 1, are considered where estimates are conducted after (or without) a stepwise selection procedure on the set of explicative variables (viz, attributes). The results of Table 1 consider first the case database with only 19 attributes relevant to our problem (referred to as *before adding of 50 random factors*). Then, 50 random attributes are added and the computations of both sub-scenarios are once again repeated.

**Table 1:** List of the attributes and weights used by the *K*-Nearest Neighbours algorithms before and after adding the 50 random attributes, and before and after stepwise selection of the case description attributes.

		Before adding of 50 random factors		After adding of 50 random factors	
		*Before attribute selection*	*After attribute selection*	*Before attribute selection*	*After attribute selection*
Social and demographic factors	Sex	0.0%	–	0.2%	–
	Age*	65.4%	68.8%	12.2%	23.9%
	Current occupation*	2.5%	2.7%	1.3%	1.3%
Clinical and biological factors	diabetes (type 1 or 2)	1.0%	–	2.7%	2.5%
	Hypertension	5.2%	5.1%	5.2%	4.8%
	Chronic respiratory failure	0.4%	–	2.4%	1.9%
	Chronic heart failure	2.0%	–	1.3%	2.2%
	Ischemic heart disease	5.7%	7.3%	2.0%	1.3%
	Heart conduction disorder (or arrythmia)	0.2%	–	0.8%	1.2%
	Past history of malignancy	6,1%	4.5%	3.1%	4.3%
	Positive serology (HCV, HBV, HIV)	1.3%	–	1.4%	–
	Liver cirrhosis	0.9%	–	1.0%	1.9%
	Disability	2.7%	3.0%	1.5%	1.5%
	Hemoglobin (< or ≥ 11 g/dl)	0.0%	–	0.0%	-
Factors related to medical care	Ownership of nephrology facilities (private or public)	3.4%	5.9%	0.1%	–
	Institution performing transplantation	3.1%	2.8%	0.1%	–
	Hemodialysis or perotoneal dialysis*	0.0%	–	1.4%	2.6%
	Urgent or planned dialysis session*	0.0%	–	0.1%	–
	Urgent or planned first catheterization	0.0%	–	0.2%	1.8%
**Random factors**		**0.0%**	**0.0%**	**63.1%**	**48.9%**

* at the first renal replacement therapy;

† HCV: Hepatitis C Virus, HBV: Hepatitis B Virus, HIV: Human Immunodeficiency Virus.

As expected, the attributes have a different impact on the registration. Their respective impact reflects on the performance of the *K*-NN algorithm through the values of the weights of attributes. When only the 19 relevant factors are considered and without a stepwise selection procedure, the most relevant predictive factors seem to be: age, hypertension, ischemic heart disease, past history of malignancy, ownership of nephrology facilities and follow-up in institution performing renal transplantation. It is worth noting that age and past history of malignancy are the only factors with a significant Wald test value. After the stepwise selection procedure, LR kept the same eight predictive factors where age, hypertension, ischemic heart disease and ownership of nephrology facilities showed a significant Wald test value.

We can notice that the logistic regression performed in this study showed results equivalent to those described in recent literature [8], [14]. We used both medical and non-medical predictive factors of transplant registration. As mentioned in section 0.1, non-medical factors might not be relevant for clinical practice; however our main objective is to discuss the efficiency of a new computational *K*-NN and not to meet concrete decision-making applications. *Age* in this kind of application field is, with no surprise, one of the most relevant clinical factors. As it could be expected, it showed a very high weight level compared to other factors. This fact might limit the results of the study. Nevertheless, since we need to design a decision-making process that performs automatically, we decided to keep the factor *age* within the discriminating factors in LR and *K*-NN algorithms.

After adding 50 random factors, estimates from the LR and the weights of attributes showed a significant change. As a matter of fact, the weight of *age* at the first RRT, for example, decreased from 65% and 69%, respectively before and after stepwise attribute selection, to 12% and 24% in the protocol arm including the random factors. Overall, the role of both the socio-demographic factors and the factors related to medical care decreased after the introduction of random factors, while the role of clinical and biological factors remained stable. The decrease of the values of sociodemographic factors’ weights and factors related to medical care happened in favor of random factors that kept a significant weight on prediction despite the selection of the attributes by a stepwise selection procedure. As expected, adding random factors creates an artifact in the definition of the relevant factors and the course of the prediction procedure. This artefact help us assess the robustness of LR combined with K-NN algorithms which is discussed in the rest of this Section.

**Figure 2:**
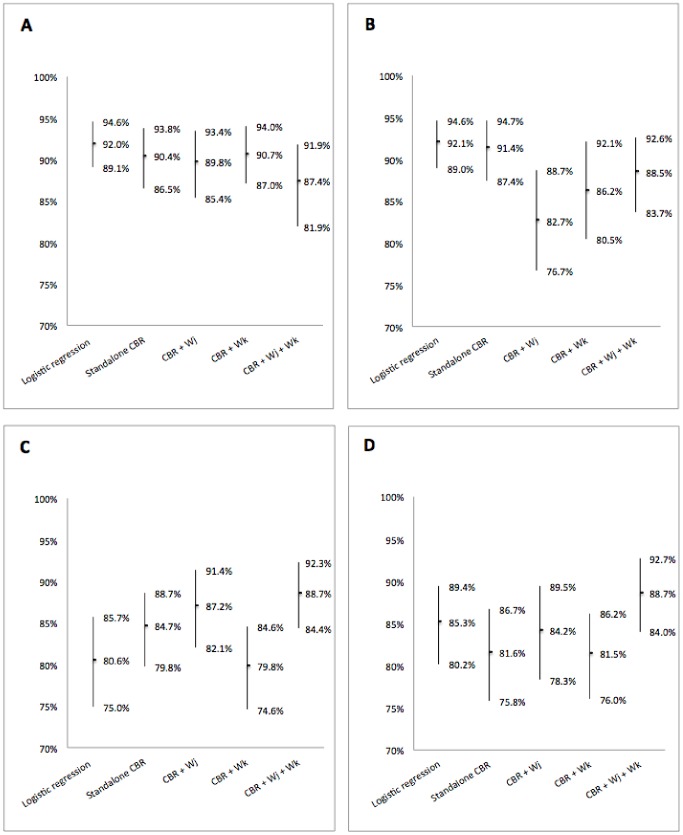
Performances of the different classification algorithms. Predictions were performed by a logistic regression, a *K*-NN algorithm (*standalone CBR*), and three combinations of the *K*-NN algorithm with the logistic regression: *CBR+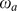
* - a *K*-NN with weighted attributes, *CBR+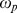
* - a *K*-NN with weighted patients, *CBR+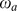
+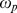
* - a *K*-NN with both weightings of attributes and patients. Performances are presented in terms of bootstrap estimates of the aera under the ROC curve with 95% confidence intervals. Prediction before adding the 50 random variables, using either the complete available attributes of the case database (**A**), or only the attributes selected by a stepwise automatic selection procedure (**B**). Prediction after adding the 50 random variables, using either the complete available attributes of the case database (**C**), or only the variables selected by a stepwise automatic selection procedure (**D**).

The Figure 2 shows the prediction results performed by the LR and the CBR methods using the *K*-NN standalone, the *K*-NN with weighting of either attributes or patients, and using the *K*-NN with weighting of both patients and attributes; respectively before and after adding 50 random attributes (as summarized in Figure 1).

First of all, we evaluate the performance of the algorithms in the ideal case with no artifact, i.e., only the 19 relevant attributes are considered. In this context, results show that predictions provided by LR and standalone CBR methods tend to be more powerful than methods combining *K*-NN and LR. This is not a surprise as both LR and *K*-NN are known to be quite efficient when the attributes are relevant. Right sub-figure in Figure 2a and Figure 2b show the performances of the tested algorithm in the ideal case with no artifact, however a pre-selection of the attributes in conducted before computing the algorithms. We notice that their performances do not significantly change except for the algorithm referred to as *CBR+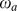
* (viz, *K*-NN with weighted attributes). As a matter of fact, we notice that this latter suffers a significant performance decrease. Since a stepwise selection of the attributes is conducted before launching the algorithm, i.e., before weighting the attributes and computing the *K*-NN algorithm, we can conclude that the stepwise attribute selection might discard some of the attributes that seem to have a significant impact when the attributes are weighted later.

Then, a similar evaluation is performed after adding 50 random attributes that, usually, are not considered as relevant. In such a scenario, the standalone LR and *K*-NN could suffer difficulties as the context is not optimally chosen to tune their performances. This is indeed observed in Figure 2c and Figure 2d where the performances of standalone LR and *K*-NN degrade significantly. One of the most interesting results through out Figure 2 is the robustness of the combination of LR and CBR when both attributes and patients are weighted. As a matter of fact, in all scenarios, with or without artifact, with or without stepwise attribute selection, the algorithm referred to as *CBR+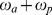
* performs in a consistent way. It provides for all scenarios a prediction rate around 88%; whereas all other algorithms, tested in this paper, seem to suffer at one point or another. This robustness offers a performance guaranty. This latter might prove to be less efficient than others in some specific scenarios, however since in realistic scenarios it is usually impossible to tell *a priori* wheather there is an artifact or not, choosing the algorithm that combines both weighted attributes and weigthed cases seems to be a cautious choice.

## Discussion

Pattern recognition in the present study used logistic regression and *K*-NN algorithm, as they represent classical methods respectively in biomedical and CBR domains, and thus it could be interesting to combine them for medical CBR systems. Nevertheless, although logistic regression analyses are widely used in medical research, it is more commonly reserved for determining prognostic factors than for predicting disease. In addition, *K*-NN is known to be slightly unstable, which could probably lead to inconsistencies in the individual estimations and predictions.

A number of other data mining and statistical methods have been applied in the medical field to assist discriminative tasks and binary classifications, as diagnosis decision-making [19], [20]. However, classification and predictive accuracy remain not-sufficient to justify a routine practice, which often results in developing and using more and more sophisticated techniques (e.g. *Artificial Neural Network, Bayesian Network, support vector machine, adaptative regression models…*). Another emerging approach is rather to include more information into classification rules, and to combine simple classifiers in order to improve predictive ability of the classification ensemble (e.g. *bagged and boosting methods*) [21], [22]. Most theoretical analysis confirms superiority of sophisticated or combinative methods, however, in real analysis of medical data, performance of more simple methods is often at least comparable [23]. And considering clinical interpretation and applicability, simple models are often more appropriate than complexe ones. It is entirely in that spirit that the methods of the present paper have been thought: a simple and well-known method, as the LR, has been used to fine-tune a simple and explicit methodology, as the *K*-NN algorithm into a CBR system.

To our knowledge, no study evaluates prediction of access to the renal transplant waiting list by a LR. Bayat *et al* invested the issue in two recent publications using a Bayesian Network and a Classification And Regression Tree method [10]. They do not present any AUCs, thus it is not possible to directly compare their results with ours. However, they conclude both methods have very high predictive performances and age is the most important factor for predicting access to the waiting list, which is coherent with our results. In another domain, Chuang compared several classifiers including LR and CBR methods to predict presence of liver disease [24]. For the author, results related to CBR methods testify to the solid diagnosis capacity of CBR in examining healthy data. Our results support this conclusion since we have shown that CBR method present predictive performances equivalent to those obtained by LR. This paper shows however that it is true only if the considered attributes are well chosen and reliable regarding the problem to solve.

Nugent *et al* presented the first association between CBR and LR in 2009 with a methodology called KLEF for *Knowledge - Light Explanation Framework* [25]. The method describes how gaining high-level knowledge by a *top-down* mechanism using logistic regression. LR is used a posteriori to define one nearest neighbour from cases retrieved by a *K*-NN algorithm. LR in the present study was used differently. As a matter of fact, the logistic model was directly fitted from the overall knowledge database. Information from LR was a direct contribution to compute similarity measures and classification probabilities. This latter approach is described by Stahl *et al* as a *bottom-up* mechanism [26].

To the best of our knowledge, only two publications describe methods similar to our hybrid approach. The first one is applied to breast cancer diagnosis (Huang *et al* [27]) and the second one is applied to the diagnosis of liver disease (Chuang [24]). In Chuangs paper, CBR methodology is different from the one applied in the present study. As a matter of fact, similarity measures are performed separately for cases with and without liver disease. In Huangs paper, similarity computation is performed through a *K*-NN algorithm, but LR is only used for defining the most relevant factors and to compute attribute weights. In the present study, LR is also used to perform attribute selection and attribute weighting. However, we proposed in addition to introduce Pearson residuals to weight the cases in the design of our *K*-NN algorithm. In our opinion, Pearson residuals based case weighting help, with attribute weighting, to the cases’ description and specification when defining problem-specific knowledge [6]. Thus, the model built by LR defines an archetype of registered and not registered patients in the knowledge database, and LR residuals reflect the adequacy of each patients with regard to the archetype. Relying only on regression coefficients or stepwise selection to define the cases as well as the problem utility would consider that all patients match perfectly the LR archetype. We know for a fact that it is not true. Hence, computing specific weights for each case, relying on LR residuals, appears as an attempt to correct of that approximation. To the best of our knowledge, this is the first time that such an approach is discussed in the literature.

As for Chuangs paper, the author points out classification improvements relying on Hybrid CBR approach compared to a standalone CBR. Huang’s publication also compares several kinds of hybrid approaches: a neural network with or without fuzzy logic and two hybrid CBR systems, one combining CBR with a decision tree and one combining CBR with LR. The neural networks show superior performances, but the authors emphasized rapidity of cases retrieval and the more easily interpretable results of CBR methodology. In the present study, the CBR hybrid approaches did not show significant improvements for patient classification, compared to standalone CBR approach. However, the hybrid CBR system combing both attribute weighting and case weighting seems to be very robust to artifacts in the database that might occur in all realistic scenarios. From our point of view, this interesting observation provides new perspectives for future CBR system, particularly for integrating CBR systems into large and unspecific knowledge database such as data from electronic health records [4], [28].

Finally, we join Huang *et al*’s opinion as we believe that CBR is an explicit problem solving methodology. We believe that an association between LR and CBR systems improves comprehensiveness of problem-solving processing. This latter provides the users with more reliable information about relevant decision factors and case utility. Thus, the integration of bio-satistical analyses, widely used in the medical research, may also help in the dissemination and development of CBR decision support for medical practice.

## Conclusions

In the paper, we presented and detailed different ways of coupling a *K*-NN algorithm and a logistic model. We have used logistic modeling in order to perform selection and weighting of cases’ features, and a new methodology have been proposed to define cases’ utility using residuals of the logistic regression. The logistic regression herein worked as an automated bottom-up procedure to define problem-specific similarity measures, and we have showed that it could improve algorithms of case retrieval and optimize reuse of cases, and at the same time it could improve CBR performance and robustness, especially when facing unspecific knowledge such as data coming from clinical care directly.

Reuse of medical data for secondary purposes, such as translational biomedical research, public health and healthcare quality improvment, provides large and interesting perspectives for medical informatics. Many initiatives have already explored solutions for integrating clinical data (e.g. caBIG [29], BRIDG [30], I2B2 [31], STRIDE [32], R-oogle [33]), and several recent projects are of interest for data warehousing, sharing and analysing of heterogeneous clinical dataset (e.g. *DebugIT* to improve detection and elimination of bacteria [34], *EU-ADR* to improve detection of adverse drug events [35] and *EHR4CR* to improve clinical trials recruitment [36]). Recognition pattern algorithm and CBR are promising methods for the secondary use of data, for instance in an hospital information system by automatically identifying new eligible patients to the clinical trials going on at the hospital (ASTEC project [37]), or by automatically detecting new healthcare-associated infections [38].

Nevertheless, althought electronic information systems and data warehouse offer opportunity for secondary use of data, it still is challenging in practice to reuse data [39], and in our opinion, it still is necessary in medical field to apply methods manually supervised to lead and control automated medical decision support. In addition, in medical field, the process of clinical care are so complexe that it still appears necessary to provide tools that could provide to the users a better understanding of the decision-making process, and tools that allow adaptating the decisions to the varying clinical context.

In our opinion, CBR integration in medical decision support is not only dependent of the ability to introduce practical and patient-oriented data elements in problem-solving procedure, even though they are essential for decision making in medical practice, but also on their ability to be fully integrated into medical reasoning processes. The hybrid approach we suggested and discussed, could thus also help to meet both requirements.
